# High-capacity assay to quantify the clonal heterogeneity in potency of mesenchymal stem cells

**DOI:** 10.1186/1753-6561-5-S8-O14

**Published:** 2011-11-22

**Authors:** Kim C O’Connor, Katie C Russell, Donald G Phinney, Michelle R Lacey, Bonnie L Barrilleaux, Kristin E Meyertholen

**Affiliations:** 1 Department of Chemical and Biomolecular Engineering, Tulane University, New Orleans, LA 70118, USA; 2 Scripps Research Institute, Jupiter, FL, 33458, USA

## Background

The regenerative capacity of mesenchymal stem cells (MSCs) is contingent on their content of multipotent progenitors [1]. Despite its importance to the efficacy of MSC therapies, the clonal heterogeneity of MSCs remains poorly defined. To address this deficiency, the current study presents a novel high-capacity assay to quantify the clonal heterogeneity in MSC potency and demonstrates its utility to resolve regenerative properties as a function of potency. Human bone marrow was the source of MSCs in this study. The versatility and accessibility of marrow-derived MSCs make them a standard for many therapeutic applications.

## Materials and methods

Primary MSCs were harvested from the iliac crest bone marrow of healthy adult volunteers and cultured as previously described [2]. The in vitro assay developed for this study utilizes a 96-well format to (1) clone fluorescent MSCs stained with CellTracker Green by limiting dilution, (2) generate matched clonal colonies, (3) differentiate 3 matched colonies per clone to quantify trilineage potential to exhibit adipo-, chondro- and osteogenesis as a measure of potency, and (4) cryopreserve the 4th matched colony of each clone in situ in an undifferentiated state for future use. Clones of known potency were evaluated for their colony-forming efficiency as a measure of proliferation potential [3]. Expression of the heterotypic cell adhesion molecule CD146 on the surface of MSC clones was measured with flow cytometry.

## Results

All eight categories of trilineage potential were detected in human marrow MSCs. Multipotent MSCs had a higher proliferation potential than lineage-committed MSCs. Tripotent clones formed colonies with a median efficiency of 50%, as compared with 14% and 1% for bi- and unipotent clones, respectively (*p* < 0.01). Likewise, colonies that formed from tripotent clones had the largest median diameter. CD146 may be a biomarker of MSC potency. Histograms of fluorescence intensity from pooled tripotent clones labeled with anti-CD146 antibody shifted to higher CD146 expression relative to the parent MSC preparation from which the clones were generated; whereas, the histograms for parent MSCs and their unipotent progeny were similar. In particular, the mean fluorescence intensity of tripotent clones was nearly twice the value for the parent and unipotent MSCs (*p* < 0.05).

## Conclusions

The research presented here addresses a basic deficiency in stem cell technology by developing a quantitative and high-capacity assay to characterize the clonal heterogeneity of MSC potency. The data suggest a complex hierarchy of lineage commitment in which proliferation potential and CD146 expression diminish with loss of potency. The capacity of multipotent MSCs for ex vivo expansion and their differential expression of a potential potency marker will facilitate rapid production of efficacious MSC therapies with consistent progenitor content. The assay has numerous basic research and clinical applications given the importance of heterogeneity to the therapeutic potential of MSCs.
